# Expression of Oncostatin M in Early Gastric Cancer and Precancerous Lesions

**DOI:** 10.1155/2019/3616140

**Published:** 2019-12-01

**Authors:** Jihua Shi, Xue Xu, Jun Du, Haimeng Cui, Qingfeng Luo

**Affiliations:** ^1^Department of Gastroenterology, National Center of Gerontology, Beijing Hospital, Beijing 100730, China; ^2^Department of Pathology, National Center of Gerontology, Beijing Hospital, Beijing 100730, China

## Abstract

**Objective:**

To detect the expression of the Oncostatin M (OSM) gene and encoded protein in the mucosal epithelium of chronic gastritis, intestinal metaplasia (IM), low-grade intraepithelial neoplasia (LGIN), high-grade intraepithelial neoplasia (HGIN), early gastric cancer (EGC), and advanced gastric cancer (AGC) samples and to explore the correlation and clinicopathological significance of OSM expression in the process of gastric carcinogenesis.

**Methods:**

The expression levels of OSM in chronic gastritis, IM, LGIN, HGIN, EGC, and AGC samples were detected by gene chip, real-time quantitative PCR, and immunohistochemical methods. The expression levels of OSM in the gastric mucosa were analyzed, and its correlation with clinical pathology was studied.

**Results:**

The expression level of OSM in gastric HGIN and EGC tissues was significantly higher than that in LGIN tissues based on expression profiling (*P* < 0.001). The expression of the OSM gene in EGC was higher than that in HGIN (unpaired *t* test, *P* < 0.05) and LGIN (unpaired *t* test, *P* < 0.01) by qPCR. The expression of OSM in LGIN was significantly lower than that in HGIN (*P* = 0.008) and EGC (*P* = 0.044) by immunohistochemical staining. The expression of OSM in HGIN tissues was significantly higher than that in AGC (*P* = 0.007).

**Conclusion:**

Alterations in the expression of the OSM gene may be involved in the malignant transformation of the gastric mucosal epithelium. Because of the significant difference in the cancerization rate and clinical management between LGIN and HGIN, the difference in the staining intensity of OSM between LGIN and HGIN may be one of the early markers of gastric intraepithelial neoplasia.

## 1. Introduction

Gastric cancer ranks fourth among the most common malignant tumors in the world and the second leading cause of cancer death [1]. The morbidity and mortality of gastric cancer rank second and third, respectively, among malignant tumors in China [2]. Early gastric cancer (EGC) refers to lesions confined to the mucosa and submucosa, regardless of lymph node metastasis. The 5-year survival rate of early gastric cancer patients is significantly higher than that of advanced gastric cancer patients (90% and 10.7%, respectively) [3, 4]. Precancerous lesions refer to tissues with histomorphological abnormalities and malignant potential of gastric cancer. According to the WHO classification of digestive system tumors (2010 edition) [5], precancerous lesions include low-grade intraepithelial neoplasia (LGIN) and high-grade intraepithelial neoplasia (HGIN). Intestinal metaplasia (IM) is a kind of precancerous state. The canceration rate reported in the literature in the past 10 years fluctuates from 0 to 1.8% [6]. Unlike advanced-stage gastric adenocarcinoma (AGC), early gastric cancer and precancerous lesions (or conditions) often have no obvious clinical symptoms. Improving the level of early diagnosis of gastric cancer and precancerous lesions will help improve the overall survival rate in gastric cancer.

Oncostatin M (OSM) is a glycoprotein secreted mainly by activated macrophages and T lymphocytes. Oncostatin M can inhibit the proliferation of melanoma cells and is an anti-oncogene. Because of its role in the activation of the STAT3 signaling pathway, OSM participates in the regulation of the tumor immune microenvironment [7]. At present, reports on the study of OSM in the gastric mucosa are scarce. This study explored the expression and clinical significance of OSM during the process of continuous development of gastric mucosal lesions and provided clues for the study of molecular markers.

## 2. Materials and Methods

### 2.1. Clinical Data

From January 2015 to January 2018, 110 patients, including 59 males and 51 females, with an average age of 58.6 years (39-82 years), completed endoscopic submucosal dissection (ESD) treatment or underwent surgery at the Department of Gastroenterology, Beijing Hospital. ESD and surgical specimens were taken every 2 mm and embedded into wax blocks. Pathological diagnosis was based on the WHO classification of digestive system tumors (2010 edition) and hematoxylin-eosin (HE) staining. Regardless of lymph node metastasis, early gastric cancer was defined as cancer limited to the mucosa and submucosa, and advanced gastric cancer was defined as cancer infiltrating beyond the submucosa. According to the pathological diagnosis of HE staining, 65 chronic gastritis, 45 intestinal metaplasia, 24 low-grade intraepithelial neoplasia, 46 high-grade intraepithelial neoplasia, 33 early gastric cancer, and 18 advanced gastric cancer samples were selected. The general clinical and pathological information is shown in Table 1. This study was approved by the Ethics Committee of Beijing Hospital and was in strict accordance with the World Medical Association Declaration of Helsinki Ethical Principles for Medical Research (Association 2013).

The tissue expression level of the OSM gene was detected in two parts. First, the tissue expression level of the OSM gene in gene chip data (GEO accession: GSE55696) was detected by searching NCBI GEO datasets (search terms: gene expression profiling, gastric precancerous lesions). Second, real-time quantitative PCR was performed in samples from the patients in this study. The expression level of the OSM gene in tissues was measured by qPCR.

### 2.2. Antibodies and Reagents

TaqMan Gene Expression Assay and TaqMan Gene Expression Master Mix were purchased from Ambion, ABI; xylene from Beijing Yili Fine Chemicals Company; phosphate-buffered saline (PBS), citrate buffer, and diaminobenzidine (DAB) from Beijing Zhongshan Jinqiao Biotechnology Company; and sheep anti-rabbit IgG (second antibody) from Origene Company, USA. Anti-oncostatin M (rabbit antibody, primary antibody) was purchased from Novus Company, USA.

### 2.3. TaqMan® Real-Time PCR

For real-time quantitative PCR (qPCR), SuperScript™ II Reverse Transcriptase and TaqMan® Gene Expression Assays (Applied Biosystems) were used. First, 500 ng of mRNA was reverse transcribed, and cDNA was produced. PCR was carried out in a total of 20 *μ*l reaction mixture (9 *μ*l of cDNA and H_2_O, 1 *μ*l of TaqMan® Assay, and 10 *μ*l of TaqMan® Gene Expression Master Mix). A 96-well plate (MX3005P™, Stratagene) was used for real-time PCR. The PCR program was initiated for 2 min at 50°C and 10 min at 95°C before 45 thermal cycles, each for 15 seconds at 95°C and 1 min at 60°C. Data were analyzed according to the comparative Ct method, and POLR2A (polymerase (RNA) II (DNA directed) polypeptide A) was chosen as the reference gene. OSM and POLR2A levels in each sample were detected in the same plate and were analyzed as follows: −△CTOSM = –(CTG0S2 − CTPOLR2A). As for immunohistochemistry staining, we used a polyclonal antibody against OSM (Novus, CO, USA; dilution 1 : 150). First, paraffin blocks were cut into 3 *μ*m thick sections, deparaffinized in xylene, and rehydrated in a descending ethanol-to-water gradient concentration. Endogenous peroxidase was blocked by exposure to 3% H_2_O_2_ for 10 min. For antigen retrieval, sections were subjected to boiling in a microwave in citrate buffer (pH = 9.0) for 5 min. After cooling to room temperature, the sections were incubated with a polyclonal antibody against OSM at 4°C overnight and incubated for 60 min at room temperature. Finally, tissue sections were subjected to chromogen reaction with 0.02% diaminobenzidine and were counterstained with hematoxylin. The positive control was from human seminiferous duct cells in testis tissues.

All immunostained slides were evaluated independently by two observers. Evaluators were blinded to the corresponding clinical data. The percentage of OSM-positive cells was scored as follows: 0-25%: 0, 26%-50%: 1, 51%-75%: 2, and >75%: 3. The intensity of cytoplasmic staining was scored as follows: no staining = 0, mild = 1, moderate = 2, and strong = 3. For statistical analysis, the percentage and intensity scores were multiplied to obtain a composite expression score (0-9). A composite score of 0-1 was classified as negative, 2-6 was ranked as weakly positive, and 7-9 was ranked as strongly positive.

### 2.4. Statistical Methods

GeneSpring software GX 12.6 was used for data analysis. After scanning and extraction, data were imported into GeneSpring software (GX 12.6) for normalization and principal component analysis (PCA). Differentially expressed genes between different groups were screened, and an unpaired *t* test was used to compare two groups, which were corrected by Benjamini and Hochberg FDR (false discovery rate). SPSS 18.0 software was used for statistical analysis of other data. Categorical variables were tested by chi-squared tests between groups. Unpaired *t* test and one-way ANOVA were used for numerical variables. The LSD (least significant difference) test was used for multiple comparisons in variance analysis. The Kolmogorov-Smirnov test was used for the normality test. Pearson's chi-squared test was used for categorical variables as the statistical significance criterion, *α* = 0.05.

Real-time quantitative PCR data (−ΔCTG0S2 and −ΔCTOSM) were analyzed using SPSS version 18.0. Independent-samples *t* tests and one-way ANOVA were performed. The SNK (Student-Newman-Keuls) test and LSD (least significant difference) test were performed, and the LSD test results are shown below. *P* < 0.05 was considered statistically significant. For immunohistochemistry staining, statistical analysis was performed using the chi-squared test. A *P* value of less than 0.05 was considered significant.

## 3. Results

### 3.1. General Clinical Information

### 3.2. The Expression of OSM in LGIN, HGIN, and EGC Samples Displayed a Gradual Increasing Trend at the Level of mRNA

#### 3.2.1. OSM Expression Pattern in Gene Chip Data

The expression level of the OSM gene in EGC was 8 times higher than that in LGIN (*P* < 0.001 after multiple correction of unpaired *t* test FDR) and that in HGIN was 4 times higher than that in LGIN (*P* < 0.001 after multiple correction of unpaired *t* test FDR). The expression level of the OSM gene in EGC was twice as high as that in HGIN (*P* > 0.05 after multiple correction of FDR by unpaired *t* test). Although there was no significant difference between these levels, the expression of HGIN to EGC increased significantly with the progression of lesions. Similarly, the expression level of the OSM gene in LGIN was 1.6 times higher than that in chronic gastritis (*P* > 0.05 after multiple correction of FDR by unpaired *t* test); although there was no significant difference, with disease progression, the expression level of the OSM gene increased gradually from chronic gastritis to LGIN, as shown in Table 2.

**Figure 1:**
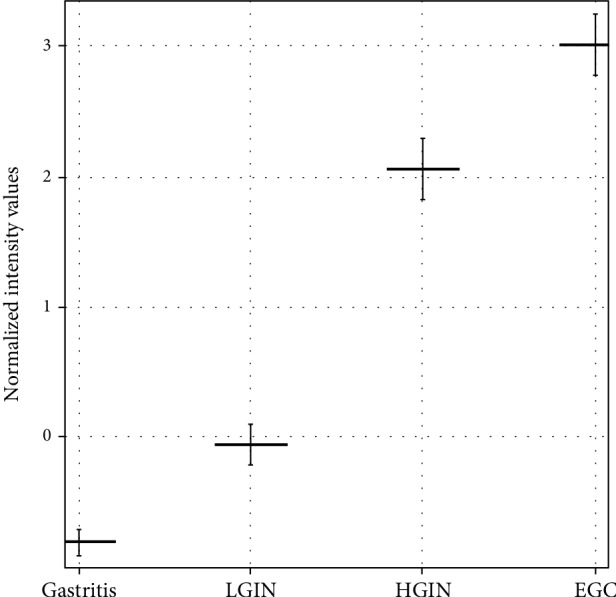
Overexpression of the OSM gene in HGIN and EGC based on gene chip analysis.

With the progression of gastric mucosal lesions, the expression level of OSM in chronic gastritis, LGIN, HGIN, and EGC gradually increased, and there was a significant difference in the expression level of mRNA among EGC, HGIN, which has higher malignant potential, and LGIN, which is relatively benign, as shown in Figure 1.

The expression level of the OSM gene in gastric HGIN and EGC tissues was significantly higher than that in LGIN tissues, but there was no significant difference between HGIN and EGC. (The expression level of the OSM gene is represented by the mean + standard error.)

#### 3.2.2. Expression Patterns of OSM Gene in Real-Time Quantitative PCR in Tissue Samples

**Figure 2:**
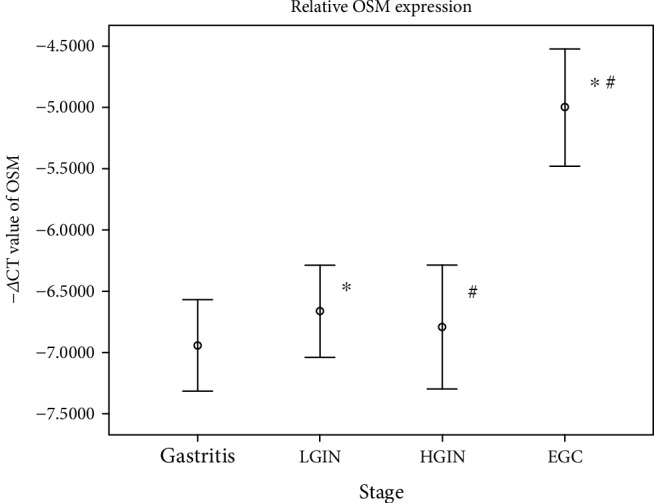
The expression level of the OSM gene was verified by qPCR in a group of gastric tissue samples. −△CT_OSM_ indicated the expression level of the OSM gene, and the results were represented as the mean ± standard. The values of −△CT_OSM_ among the four groups were subjected to variance analysis, and the homogeneity test of variance revealed *P* = 0.843, with homogeneous variance. ^∗^Significant differences in the mean values between the LGIN and EGC groups (unpaired *t* test, *P* = 0.008); ^#^significant differences in the mean values between the HGIN and EGC groups (unpaired *t* test, *P* = 0.014).

In our gastric mucosal tissue samples, the expression of the OSM gene in EGC was significantly higher than that in HGIN (unpaired *t* test, *P* < 0.05) and LGIN (unpaired *t* test, *P* < 0.01), as shown in Figure 2.

In conclusion, the expression of the OSM gene in the gastric mucosa was higher in early gastric cancer than in precancerous lesions (HGIN and LGIN). Combined with GO enrichment analysis of gene chip expression profiles and our real-time PCR analysis of OSM gene expression levels in tissues, OSM gene-related immune response function may play an important role in the early stages of gastric cancer.

### 3.3. Immunohistochemical Expression of the OSM Gene in Gastric Cancer and Precancerous Lesions

**Figure 3:**
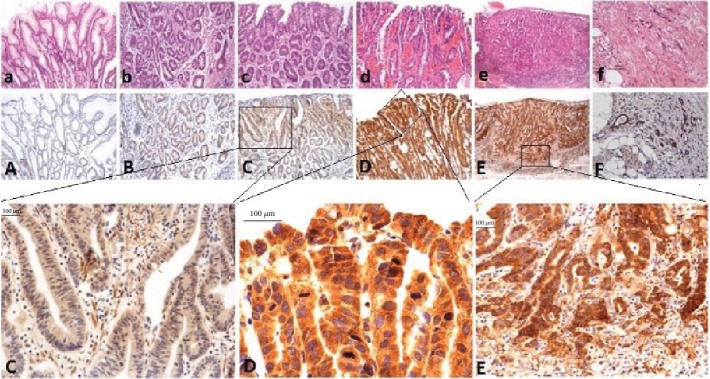
Immunohistochemical staining of OSM in different gastric mucosal lesions (OSM shows cytoplasmic staining). Panels (a) to (f) show HE staining sections corresponding to (A) to (F), respectively. Panel (A) shows normal gastric mucosa: OSM expression is negative (bar represents 100 *μ*m). Panel (B) shows intestinal metaplasia gastric mucosa: OSM expression is weakly positive (bar represents 100 *μ*m). Panel (C) shows low-grade intraepithelial neoplasia gastric mucosa: OSM expression is moderately positive (bar represents 100 *μ*m). Panel (D) shows high-grade intraepithelial neoplasia gastric mucosa: OSM expression is strongly positive (bar represents 100 *μ*m). Panel (E) shows early gastric cancer mucosa: OSM expression is strongly positive (bar represents 100 *μ*m). Panel (F) shows advanced gastric cancer mucosa: OSM is moderately positive (bar represents 100 *μ*m).

Immunohistochemical results showed that OSM-positive signals were localized in the cytoplasm as brown and yellow granules. The expression of OSM in all gastric mucosal tissues was weakly positive (OSM < 6 and < 2) in 65.8% (*n* = 150) and strongly positive (OSM > 6) in 17.5% (*n* = 40) of samples. In chronic gastritis mucosa (*n* = 65), only 58.5% of the tissues showed weakly positive expression, none showed strong positive expression, and almost half of the cases had no OSM expression. In intestinal metaplasia mucosa (*n* = 45), most cases (91.1%) showed weakly positive expression. In low-grade intraepithelial neoplasia (*n* = 22), the staining intensity of OSM increased further, with a strong positive expression accounting for 13.6% of samples, but weak positive expression was still dominant (72.7%). In high-grade intraepithelial neoplasia (*n* = 46), OSM was stained completely, and strong positive expression was detected in 39.1%, while weak positive expression was detected in 60.9% of samples. In early gastric cancer (*n* = 33), the staining intensity gradually increased, with strong positive expression accounting for 45.5% and weak positive expression accounting for 48.5% of samples. With the development of malignant tumors, the staining intensity of OSM decreased. Strong positive expression accounted for only 17.6% of samples in advanced gastric cancer (*n* = 17), while weak positive expression accounted for 64.7%. See Figure 3 and Table 3 for details.

Statistical analysis showed that the expression of OSM in gastritis tissues was significantly lower than that in intestinal metaplasia, LGIN, HGIN, EGC, and AGC tissues; the expression of OSM in intestinal metaplasia tissues was significantly lower than that in HGIN, EGC, and AGC tissues; the expression of OSM in LGIN tissues was significantly lower than that in HGIN and EGC tissues; the expression of OSM in HGIN tissues was not significantly different from that in EGC tissues but significantly higher than that in AGC tissues; and the expression of OSM in early and late gastric cancer tissues was not significantly different. See Table 3.

In conclusion, from gastritis (0%) to intestinal metaplasia (2.2%) to LGIN (13.6%) to HGIN (39.1%) to EGC (45.5%), the rate of strong positivity for OSM expression in the gastric mucosa increased gradually. However, from EGC (45.5%) to AGC (17.6%), with the progression of gastric cancer, the rate of strong positivity for OSM expression decreased gradually.

## 4. Discussion

OSM is a cytokine belonging to the interleukin-6 (IL-6) family. It can activate JAK-STAT [8], the PI3K/AKT signaling pathway [9], MAPKs, and the Ras signaling pathway [10] in conjunction with other cytokines of the IL-6 family. OSM can induce leukocyte adhesion and chemotaxis, chemokine production in endothelial cells, and promote inflammation [10]. In this regard, its role in inflammatory bowel disease have been well detected [11]. At the molecular level, OSM can directly or indirectly participate in insulin resistance [12] and muscle stem cell induction [13]. What is more, it may involve in cardiac fibrosis [14] and liver fibrosis [15] via macrophage. OSM promotes the plasticity of cancer cells through synergistic STAT3-SMAD3 signal transduction [16]. As for tumorigenesis, OSM can promote the development and metastasis of tumors in vivo [17]. Studies have shown that OSM promotes the invasion and angiogenesis of endometrial cancer by activating STAT3 [18], which is linked with tumor cell proliferation, survival, and metastatic invasion [19]. However, on the other hand, some studies suggest that the regulation of the gastric tumor microenvironment may play a key role in the process of carcinogenesis [20, 21].

In this study, combined with the previous OSM gene research [22], gene chip and real-time quantitative PCR validation results show that the expression level of the tumor-suppressive OSM gene in tissues was higher in early gastric cancer than in precancerous lesions (HGIN and LGIN). The results of immunohistochemical staining showed that the expression of the OSM protein in EGC and HGIN tissues increased most significantly, and the positive expression of the OSM protein was higher than that in other tissues. Although there was no significant difference in OSM protein expression between HGIN and EGC, the rate of strong positive expression (45.5%) in EGC was slightly higher than that in HGIN (39.1%). In fact, this is consistent with the results of the gene expression profile chip. Although the expression levels of the OSM gene in HGIN and EGC were not identical at the level of mRNA or protein, this result supports our hypothesis that HGIN and EGC are biologically very similar and that their biological differences are not significant. Another reason that cannot be ignored is that precancerous lesions of gastric cancer, like gastric cancer, also exhibit some heterogeneity.

To explore the expression level of OSM in different gastric mucosal lesions, we found that the positive rate of OSM expression in the gastric mucosa increased gradually during the progression from gastritis→intestinal metaplasia→LGIN→HGIN→EGC, suggesting that the expression of the OSM gene may be involved in the malignant transformation of the gastric mucosal epithelium. But at the same time, its expression level from the early stage to advanced stage gradually decreased, conflicting with other studies. It was recently found that OSM promotes the proliferation, migration, and invasion of gastric cancer cells and that OSM and OSM receptor contribute to GC progression by activating STAT3/FAK/SRC signaling [23]. It also has been proved that OSM can inhibit the proliferation of many cancer cell lines, including melanoma, ovarian cancer, lung cancer, gastric cancer, and breast cancer [24], by counteracting STAT3-driven tumorigenesis, via STAT1 [25]. Taken above, there may exist an interplay between STAT3 and STAT1 regulated by OSM, and the different transformations between them may affect the progress of gastric cancer. It may be a possible explanation, but more explorations are required to identify the specific mechanism in the future.

In this study, the OSM is not only expressed in gastric mucosal epithelial cells but also in some interstitial cells, lymphocyte, plasma cells, and inflammatory cells. The overexpression of OSM contributes to the progression of gastric cancer. The increase in OSM expression in gastric cancer may be related to inflammation and the tumor microenvironment. Inflammatory/immune cells in tumors secrete cytokines. Some studies have found that the expression of IL-6 cytokine family proteins, including OSM, in cancer-related fibroblasts is approximately 100 times higher than that in normal fibroblasts [26]. Cytokines and key transcription factors activating precancerous cells, IL-6 family members, and related signaling pathways may participate in the formation of positive feedback loops during tumorigenesis and development (NF-kappa B and STAT3 are important signaling pathways of tumor immunity) [27], for example, NF-kappa B can promote the production of IL-6 family cytokines, while the IL-6 family can activate downstream STAT3 signaling pathways. Cancer-related adipose tissues can promote the progression of breast cancer through paracrine OSM and JAK/STAT3 signal transduction [28]. This study is the first to detect the expression of OSM in gastric mucosal inflammatory lesions, intestinal metaplasia, dysplasia, and gastric cancer tissues. One of the important findings is that the staining intensity of OSM is significantly higher in HGIN and EGC than in LGIN. Because of the significant difference in the cancerization rate and clinical management between LGIN and HGIN, the difference in OSM staining intensity between LGIN and HGIN may be an early marker of malignant transformation of intraepithelial gastric neoplasms. Though limited to the source of patients, our sample size was not large enough, but our results that combine with the histopathological features of the lesions showed OSM have the potential in the clinical diagnosis and treatment of atypical lesions.

## Figures and Tables

**Table 1:** General clinicopathological information of patients enrolled in the group.

(*n* = 110)	Clinicopathological characteristic	
Age (year)	qPCR test	IHC staining
(Mean ± SD)	58.63 ± 7.30	
(Range)	39-82	
Sex		
(Male : female)	59 : 51	
Gastric lesions		
LGIN	26	24
HGIN	15	46
EGC	14	33
Gastritis	20	65
Intestinal metaplasia (IM)		45
AGC		18

Illustration: qPCR and IHC partially overlapped, and there might be 2-3 lesions (such as LGIN, IM, and gastritis) in the same section of partial IHC.

**Table 2:** Statistical analysis results of differences in OSM gene expression among tissue samples on expression profiling chip.

Unpaired *t* test of OSM	*P* (correlation by FDR)	*P*	Fold change (absolute value)	Probe name	Gene symbol description
Up in HGIN vs. LGIN	0.015	<0.001	4.31	A_23_P166408	*Homo sapiens* oncostatin M (OSM), mRNA [NM_020530]
Up in EGC vs. LGIN	<0.001	<0.001	8.37		
Up in HGIN vs. gastritis	<0.001	<0.001	7.25		
Up in EGC vs. gastritis	<0.001	<0.001	14.09		
Up in EGC vs. HGIN	0.535	0.155	1.94		
Up in LGIN vs. gastritis	0.086	0.049	1.68		

**Table 3:** Expression of OSM in different types of gastric mucosal lesions.

*N* = 228	OSM < 2 (*n* = 38)	OSM ≤ 6 and OSM ≥ 2 (*n* = 150)	OSM > 6 (*n* = 40)	Pearson's chi-squared test
Gastritis (*n* = 65)^a,b,c,d,e^	27 (41.5%)	38 (58.5%)	0 (0%)	*P* < 0.001
IM (*n* = 45)^f,g,h^	3 (6.7%)	41 (91.1%)	1 (2.2%)	
LGIN (*n* = 22)^i,j^	3 (13.6%)	16 (72.7%)	3 (13.6%)	
HGIN (*n* = 46)^k^	0 (0%)	28 (60.9%)	18 (39.1%)	
EGC (*n* = 33)	2 (6.1%)	16 (48.5%)	15 (45.5%)	
AGC (*n* = 17)	3 (17.6%)	11 (64.7%)	3 (17.6%)	

^a^Chi-squared test, *P* < 0.001, significant difference compared with intestinal metaplasia (IM); ^b^chi-squared test, *P* = 0.001, significant difference compared with LGIN; ^c^chi-squared test, *P* < 0.001, significant difference compared with HGIN; ^d^chi-squared test, *P* < 0.001, significant difference compared with EGC; ^e^chi-squared test, *P* = 0.001, significant difference compared with AGC; ^f^chi-squared test, *P* < 0.001, significant differences compared with HGIN and EGC; ^g^chi-squared test, *P* < 0.001, significant difference compared with EGC; ^h^chi-squared test, *P* = 0.029, significant difference compared with AGC; ^i^chi-squared test, *P* = 0.008, significant difference compared with HGIN; ^j^chi-squared test, *P* = 0.044, significant difference compared with EGC; ^k^chi-squared test, *P* = 0.007, significant difference compared with AGC.

## Data Availability

The data used to support the findings of this study are available from the corresponding author upon request.

## References

[1] Bray F., Ferlay J., Soerjomataram I., Siegel R. L., Torre L. A., Jemal A. (2018). Global cancer statistics 2018: GLOBOCAN estimates of incidence and mortality worldwide for 36 cancers in 185 countries. *CA: A Cancer Journal for Clinicians*.

[2] Dai M., Ren J. S., Li N., Li Q., Yang L., Chen Y. H. (2012). Estimation and prediction on cancer related incidence and mortality in China, 2008. *Zhonghua Liu Xing Bing Xue Za Zhi*.

[3] Ohta H., Noguchi Y., Takagi K., Nishi M., Kajitani T., Kato Y. (1987). Early gastric carcinoma with special reference to macroscopic classification,. *Cancer*.

[4] Lazar D., Taban S., Sporea I. (2009). Gastric cancer: correlation between clinicopathological factors and survival of patients (III). *Romanian Journal of Morphology and Embryology*.

[5] Bosman F. T., Carneiro F., Hruban R. H., Theise N. D. (2010). *WHO classification of tumours of the digestive system*.

[6] Ribeiro M. D., Areia M., De Vries A. C. (2012). Management of precancerous conditions and lesions in the stomach (MAPS): guideline from the European Society of Gastrointestinal Endoscopy (ESGE), European Helicobacter Study Group (EHSG), European Society of Pathology (ESP), and the Sociedade Portuguesa de Endoscopia Digestiva (SPED). *Virchows Archiv*.

[7] Taniguchi K., Karin M. (2014). IL-6 and related cytokines as the critical lynchpins between inflammation and cancer. *Seminars in Immunology*.

[8] Demyanets S., Kaun C., Rychli K. (2011). Oncostatin M-enhanced vascular endothelial growth factor expression in human vascular smooth muscle cells involves PI3K-, p38 MAPK-, Erk1/2- and STAT1/STAT3-dependent pathways and is attenuated by interferon-*γ*. *Basic Research in Cardiology*.

[9] Smith D. A., Kiba A., Zong Y., Witte O. N. (2013). Interleukin-6 and oncostatin-M synergize with the PI3K/AKT pathway to promote aggressive prostate malignancy in mouse and human tissues. *Molecular Cancer Research*.

[10] Savarese T. M., Campbell C. L., McQuain C. (2002). Coexpression of oncostatin M and its receptors and evidence for STAT3 activation in human ovarian carcinomas. *Cytokine*.

[11] West N. R., Oxford IBD Cohort Investigators, Hegazy A. N. (2017). Oncostatin M drives intestinal inflammation and predicts response to tumor necrosis factor-neutralizing therapy in patients with inflammatory bowel disease. *Nature Medicine*.

[12] Stephens J. M., Elks C. M. (2017). Oncostatin M: potential implications for malignancy and metabolism. *Current Pharmaceutical Design*.

[13] Sampath S. C., Sampath S. C., Ho A. T. V. (2018). Induction of muscle stem cell quiescence by the secreted niche factor oncostatin M. *Nature Communications*.

[14] Abe H., Takeda N., Isagawa T. (2019). Macrophage hypoxia signaling regulates cardiac fibrosis via Oncostatin M. *Nature Communications*.

[15] Matsuda M., Tsurusaki S., Miyata N. (2018). Oncostatin M causes liver fibrosis by regulating cooperation between hepatic stellate cells and macrophages in mice. *Hepatology*.

[16] Junk D. J., Bryson B. L., Smigiel J. M., Parameswaran N., Bartel C. A., Jackson M. W. (2017). Oncostatin M promotes cancer cell plasticity through cooperative STAT3-SMAD3 signaling. *Oncogene*.

[17] García-Tuñón I., Ricote M., Ruiz A., Fraile B., Paniagua R., Royuela M. (2009). OSM, LIF, its receptors, and its relationship with the malignance in human breast carcinoma (in Situand in infiltrative). *Cancer Investigation*.

[18] Zhu M., Che Q., Liao Y. (2015). Oncostatin M activates STAT3 to promote endometrial cancer invasion and angiogenesis. *Oncology Reports*.

[19] Li N., Grivennikov S. I., Karin M. (2011). The unholy trinity: inflammation, cytokines, and STAT3 shape the cancer microenvironment. *Cancer Cell*.

[20] Correa P., Schneider B. G. (2005). Etiology of gastric cancer: what is new?. *Cancer Epidemiology Biomarkers & Prevention*.

[21] Milne A. N., Sitarz R., Carvalho R., Carneiro F., Offerhaus G. J. A. (2007). Early onset gastric cancer: on the road to unraveling gastric carcinogenesis. *Current Molecular Medicine*.

[22] Xu X., Feng L., Liu Y. (2014). Differential gene expression profiling of gastric intraepithelial neoplasia and early-stage adenocarcinoma. *World Journal of Gastroenterology*.

[23] Yu Z., Li Z., Wang C. (2019). Oncostatin M receptor, positively regulated by SP1, promotes gastric cancer growth and metastasis upon treatment with Oncostatin M. *Gastric Cancer*.

[24] David E., Guihard P., Brounais B. (2011). Direct anti-cancer effect of oncostatin M on chondrosarcoma. *International Journal of Cancer*.

[25] Pan C. M., Wang M. L., Chiou S. H., Chen H. Y., Wu C. W. (2016). Oncostatin M suppresses metastasis of lung adenocarcinoma by inhibiting SLUG expression through coordination of STATs and PIASs signalings. *Oncotarget*.

[26] Hugo H. J., Lebret S., Tomaskovic-Crook E. (2012). Contribution of fibroblast and mast cell (afferent) and tumor (efferent) IL-6 effects within the tumor microenvironment. *Cancer Microenvironment*.

[27] Grivennikov S. I., Greten F. R., Karin M. (2010). Immunity, inflammation, and cancer. *Cell*.

[28] Lapeire L., Hendrix A., Lambein K. (2014). Cancer-associated adipose tissue promotes breast cancer progression by paracrine oncostatin M and Jak/STAT3 signaling. *Cancer Research*.

